# Impedance-based in vitro eye irritation testing enables the categorization of diluted chemicals

**DOI:** 10.1038/s41598-024-56191-2

**Published:** 2024-03-14

**Authors:** H. Weissinger, N. Knetzger, C. Cleve, C. Lotz

**Affiliations:** 1 Translational Center Regenerative Therapies (TLC-RT), Fraunhofer Institute for Silicate Research (ISC), Würzburg, Germany; 2 Tissue Engineering and Regenerative Medicine (TERM), University Hospital Würzburg, Würzburg, Germany

**Keywords:** Translational research, Preclinical research, Public health, Biological models, Electrophysiology, Biological techniques, Risk factors

## Abstract

Products containing chemicals with eye irritation potential need to be labeled with the respective hazard symbol. To avoid the testing of numerous dilutions of chemicals on animals, their labeling is directed by a theoretical approach. In this report, a previously described in vitro tissue model of the cornea based on human epithelial cells was used for eye irritation testing of dilutions. As a sensitive and non-destructive method to analyze the barrier function of the epithelium, impedance spectroscopy was applied. Moreover, the morphology and viability of the epithelial models were assessed. We tested four chemicals that, neatly, cause severe damage to the eye: tetrahydrofuran, acetic acid, diethylethanolamine, and benzalkonium chloride. With our test method, we were able to determine the concentrations of the chemicals which are critical for the integrity of the cornea. The threshold was < 0.1% for the most and > 5% for the least toxic substance. The described test system is not only an alternative for animal models but also for the theoretical examination of the hazard potential of diluted chemicals. By using the advantages of tissue engineering and non-destructive analysis tools, we can achieve more precise and safer labeling of the eye irritation potential of products.

## Introduction

To protect the health of consumers, chemicals with hazard potential need to be labeled according to the United Nations global harmonized system (GHS). Among the health risks induced by chemicals, eye irritation has high relevance as it can lead, in the worst case, to loss of sight. The cornea as the most anterior layer of our eye is responsible for the protection of the underlying tissue. It is non-cornified to allow the transmission of light to the retina, thus it is more sensitive than the similar, but cornified epithelium of the skin. The cornea consists of a stratified epithelium, the Bowman’s membrane, which is located directly below the epithelium, a stroma, and an endothelium on top of the Descement’s membrane. Chemicals can cause reversible or irreversible damage to the cornea. Depending on the severity of the damage, the GHS classifies chemicals in category 1 (serious eye damage), category 2 (eye irritation), or no category (no irritation). The eye irritation potential of chemicals is currently determined by the in vivo Draize eye test, for which the chemical is applied to the eye of a rabbit^1^. Due to ethical concerns, low reproducibility, and low accuracy of this test, new approach methodologies based on 3D in vitro models are developed and adopted by the Organisation for Economic Co-operation and Development (OECD)^2^. Models based on human cells are advantageous in predicting hazards, as toxic effects can vary between species^3^. While more complex models that include several layers of the cornea can be useful for other research purposes, regulatory questions are typically pursued in epithelial models. To assess damages on epithelial models, several methods are applicable, including histology or viability assays. Further, impedance spectroscopy is a quantitative, non-destructive method to measure the barrier function^4^.

**Figure 1:**
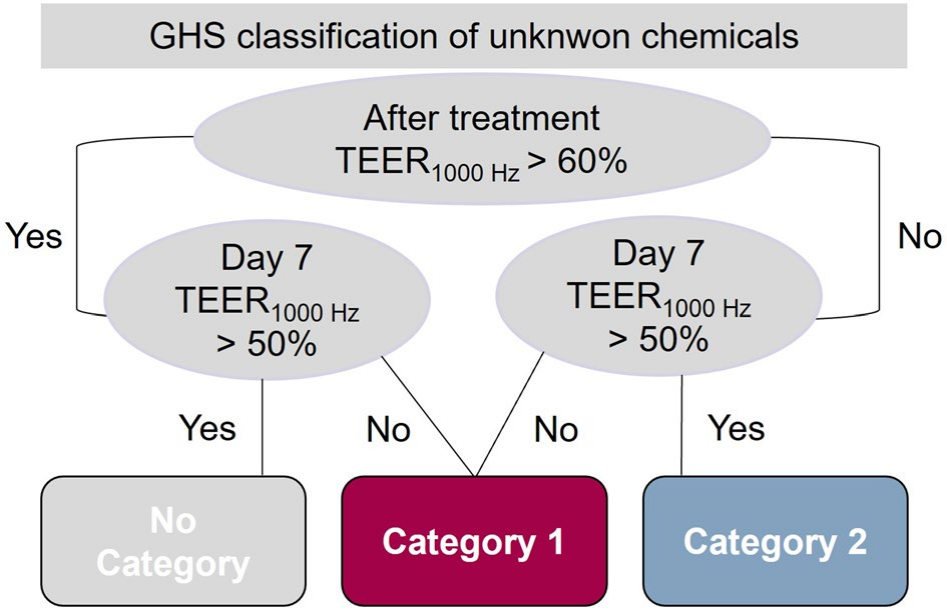
The prediction model used to classify unknown chemicals according to their GHS category. The classification is based on the membrane barrier functionality of TEER_1000 Hz_ (in %) normalized to the control on day 0 after application and on day 7. The decision is made on two thresholds: TEER_1000 Hz_ > 60% on day 0 after treatment and TEER_1000 Hz_ > 50% on day 7. Adapted from C. Lotz et al.^5^.

The reconstructed cornea-like epithelium (RCE) is based on primary human cells and has great potential to mimic the human cornea in vitro. An eye irritation test (EIT) based on the RCE model and repeated impedance spectroscopy allows the categorization of chemicals in all three GHS categories^5^ (Fig. 1). For this prediction model, the barrier function of the models is tested directly after EIT and on day 7 after EIT. Substances causing a reduction of the TEER_1000 Hz_ values to < 60% of the negative control directly after EIT and to < 50% at day 7 are categorized as GHS-category 1 substances. If the TEER_1000 Hz_ values are > 60% after EIT, but < 50% at day 7, the tested substance is also classified as a category 1 substance. If the TEER_1000 Hz_ values are < 60% after EIT but are reestablished to > 50% at day 7, a category 2 substance is predicted. Substances causing no reduction of TEER_1000 Hz_ values to < 60% after EIT or < 50% at day 7 fall in no category.

In many products, chemicals are present in dilution or mixture with other substances. Hence, the potential for eye irritation can be altered and the product might fall into another GHS category than the neat substance. Since 2015, mixtures containing diluted chemicals must be labeled according to the *“Classification, Labeling and Packaging”* (CLP) Regulation by the European Union^6^. The classification of dilutions is made based on general concentration limits for the respective category and if available, existing data for the diluted substance. In addition, data for similar chemicals can be considered. The theoretical classification system is not only complex but might also be less safe for the consumer which underpins the necessity for new animal-free in vitro test systems. This report shows how the RCE model in combination with impedance spectroscopy can serve as a test system for the eye irritation potential of diluted chemicals.

As test substances, four chemicals of GHS category 1 were chosen. They cover a range of different chemical properties and modes of action, and were therefore selected to represent the chemicals of this category. In addition, the selected substances cover different drivers of classification such as corneal opacity (CO), iris redness (IR) and conjunctival redness (CR)^7^. Tetrahydrofuran (THF) is known as a solvent, but its derivates also demonstrated potential as human immunodeficiency (HIV) protease inhibitors^8,9^. Acetic acid (AA) is used in the food industry as a preservative and acidity regulator, has antioxidant and antimicrobial effects, and is present in vinegar in concentrations between 4 and 8%^10^. Diethylethanolamine (DEEA) often serves as a chemical precursor in industry, has anti-corrosive properties, and potently absorbs CO_2_^11,12^. Benzalkonium chloride (BAC) is a component of many pharmaceuticals, including eyedrops, and acts as a preservative due to its antifungal and antibacterial properties^13^. The substances were tested in 100%, 5%, and 1% dilution. As category 1 chemicals cause severe irritation, we expected a reduced toxicity only in strong dilution and therefore chose 5% and 1% as test conditions. As BAC is known to exert effects even in dilutions below 1%, we tested this chemical in 0.1% and 0.01%. Testing chemicals in dilution with the presented test system could be the future of determining the correct GHS category for potentially harmful solutions.

## Results

The test system based on RCE models could not only ensure safe and ethical eye irritation tests for neat substances but further be applicable to more complex solutions. We aimed to evaluate the effects of different diluted substances on RCE models. Therefore, the models were treated with the substances in the respective concentrations and evaluated over 11 days. Impedance spectroscopy was used on day 0, before and after application, and on days 1, 3, 7 and 11. Histology and MTT were used as destructive methods on day 7 and 11. We chose day 7 for assessing the morphology, as both acute toxic effects like cell shrinking and long-term damages like reduced growth can be seen. The viability was measured at the end of the observation period, day 11, to show the final outcome of the eye irritation.

**Figure 2:**
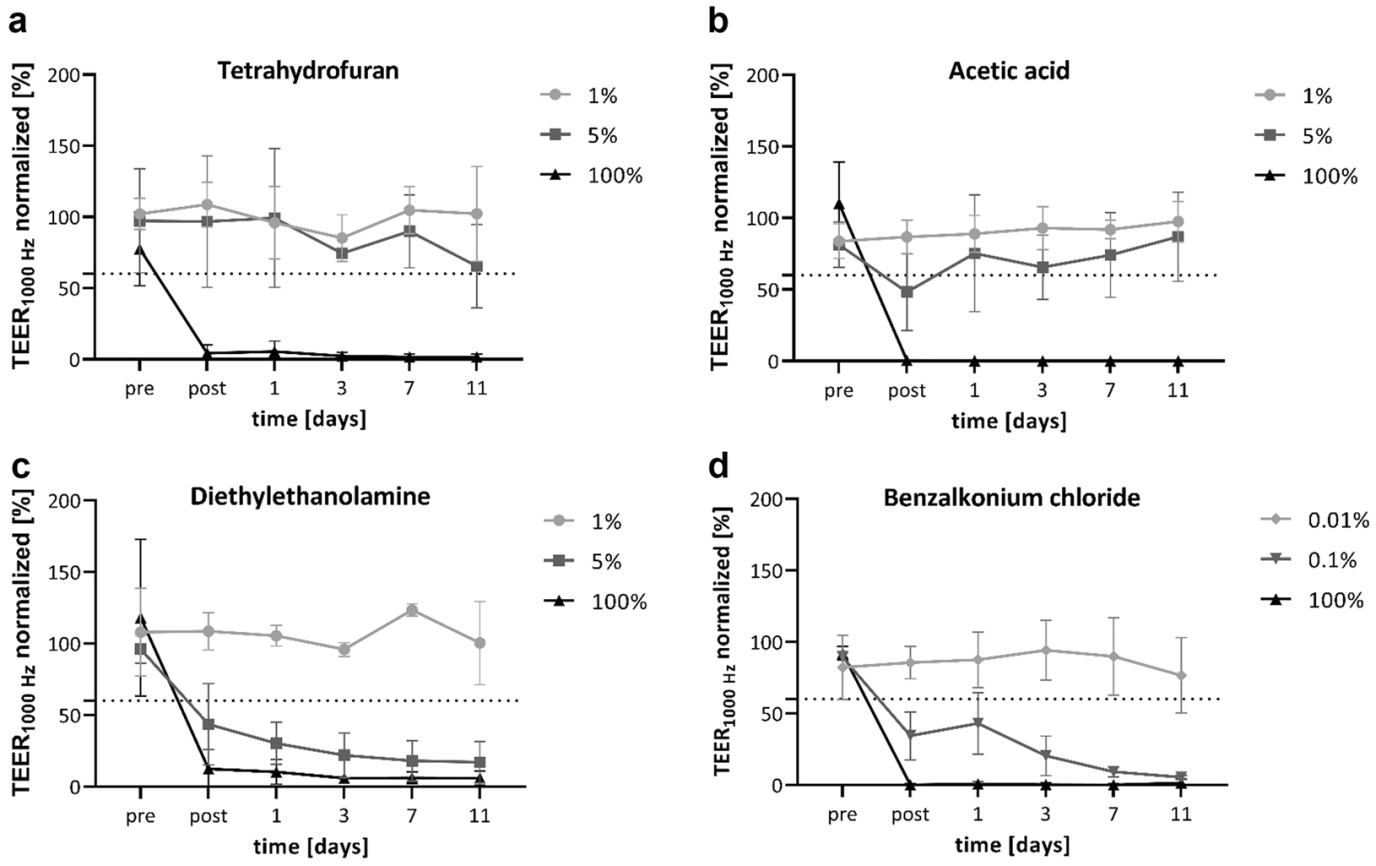
The TEER of diluted GHS category 1 substances varies between different chemicals. Impedance spectroscopy, depicted are the means with standard deviation of TEER-values at 1000 Hz normalized to the negative control. TEER-values of two models were measured for each biological replicate. Measurements were performed before and after EIT, and on day 1, 3, 7, and 11 after EIT. Biological replicates for barrier integrity measurements: n = 3.

We first set out to assess the eye irritation potentials of the diluted chemicals by impedance spectroscopy. For Tetrahydrofuran, TEER_1000 Hz_-values of 108.8 ± 12.7% for the 1% dilution and 96.7 ± 37.6% for the 5% dilution were detected after EIT. The barrier function remained intact throughout all following time points (Fig. 2a). Undiluted THF caused a significant reduction of the TEER_1000 Hz_ -values (pre vs. post: p < 0.01), and no recovery of the barrier was detected. We further applied Acetic Acid to RCE models. The barrier function was unaffected by the EIT with 1% AA, as the TEER_1000 Hz_-values were rising from 83.8 ± 9.8% before EIT to 86.7 ± 9.6% after EIT. After EIT with 5% AA, the TEER_1000 Hz_-values were reduced from 81.3 ± 12.8 to 48.3 ± 22.0%, and thus below the threshold of 60%. Interestingly, the barrier function recovered to 86.9 ± 25.4% on day 11. After the application of neat AA, TEER_1000 Hz_-values were diminished to 0.6 ± 0.4% (Fig. 2b). Diethylethanolamine was tested in the same concentrations as the aforementioned substances. We detected an intact barrier for models treated with 1% DEEA at all time points. In 5% and 100%, DEEA led to a reduction of TEER_1000 Hz_-values after EIT. While the barrier of models treated with 5% DEEA was 43.6 ± 23.1% of the negative control, models treated with neat DEEA dropped to 12.4 ± 11.1% after EIT. After treatment with 5% or 100% DEEA, the barrier of RCE models did not recover in the monitored timespan (Fig. 2c). For Benzalkonium chloride, we tested the dilutions 0.1% and 0.01% besides the neat substance. The TEER_1000 Hz_-values were largely unaffected by EITs with 0.01% BAC and rose from 82.3 ± 18.3% before EIT to 85.6 ± 9.3% after EIT. In contrast, 0.1% BAC caused a reduction of the barrier to 34.4 ± 13.7% after EIT. An incisive loss of barrier function was caused by neat BAC, and all TEER_1000 Hz_-values after EIT were below 2% of the negative control (Fig. 2d).

Comparing the effects of substances that were applied in the same concentration, we found significant differences. We focused on the TEER measurements after EIT and on day 7 for statistical analysis, as these time points are relevant to the categorization. THF, AA and DEEA were not irritative in concentrations of 1%, thus differences were not significant. BAC in 0.1% caused significantly lower TEER-values compared to all other substances in 1% (p ≤ 0.002) on day 7.

For dilutions of 5% significant differences were obtained on day 7 between AA and DEEA (p = 0.049) and between DEEA and THF (p = 0.009). As the models treated with AA could regenerate, no significance was observed between AA and THF on day 7 (p = 0.770). As category 1 substances were chosen, barrier function of all models dropped similarly after EIT with neat substances and the TEER values were comparable.

**Figure 3:**
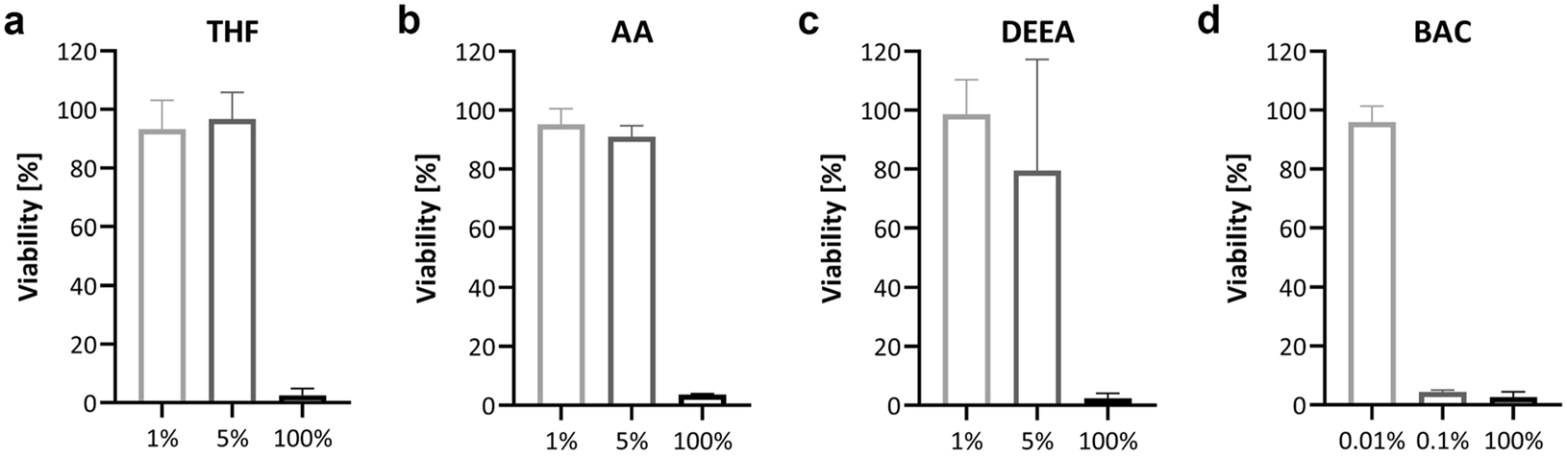
The cell viability of GHS category 1 substances in dilution varies between different chemicals. MTT assays were performed on day 11 after EIT, values are normalized to the negative control. 2 technical replicates of each RCE model were measured. Biological replicates for the MTT readout method: n = 3, means with standard deviation are shown. *THF* tetrahydrofuran, *AA* acetic acid, *DEEA* diethylethanolamine, *BAC* benzalkonium chloride.

The viability of the RCE models at day 11 after EIT was quantified by MTT assay. For THF, this resulted in values of 93.2 ± 8.1% for the 1% dilution, 96.8 ± 7.4% for the 5% dilution, and 2.6 ± 1.8% for neat THF, supporting the sustained integrity of the models treated with diluted THF (Fig. 3a). Equally in line with the impedance spectroscopy, the models treated with 1% or 5% AA were viable in MTT assay, while models treated with 100% AA were not (Fig. 3b). An unimpeded viability was visualized by MTT on day 11 after EIT with 1% DEEA, and a lack of viability was detected in models treated with neat DEEA. In comparison to the strong reduction of barrier function after the application of 5% DEEA, the viability of the models was only mildly affected (Fig. 3c). For BAC, MTT showed viable models after the application of 0.01% and no viability for models treated with 0.1% or 100% BAC, which corroborated the results of the impedance measurements (Fig. 3d).

**Figure 4:**
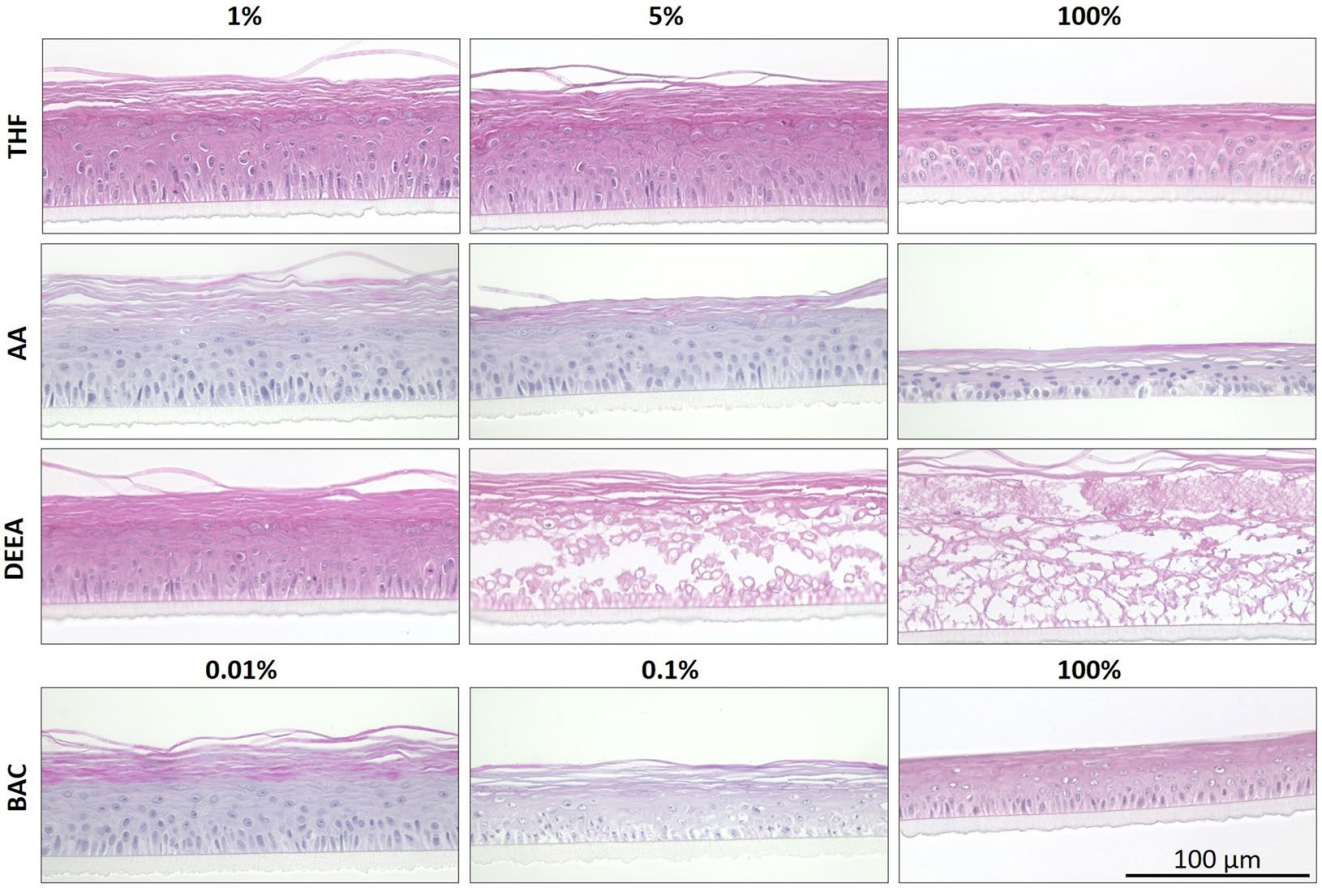
Histological analysis of GHS category 1 substances in dilution varies between different chemicals. H&E staining images of RCE models on day 7 after EIT. Biological replicates for the histological analysis were n = 3. *THF* tetrahydrofuran, *AA* acetic acid, *DEEA*: diethylethanolamine, *BAC* benzalkonium chloride.

Th effects of diluted chemicals on RCE models were moreover evaluated histologically. Application of 1% or 5% THF resulted in a stratified epithelium with differentiated cells. The neat substance led to a thin epithelium with reduced cornification (Fig. 4). In dilutions of 1% or 5%, AA did not elicit morphological changes compared to the negative control. Contrarily, the morphology of models treated with 100% AA was altered. The epithelium comprised fewer cell layers and the cytoplasm was partially disintegrated. The morphology of the models treated with 1% DEEA resembled the negative control. In dilution of 5%, DEEA caused pronounced damage to the epithelium. The cell–cell contacts were disrupted, and nuclei were pyknotic or not recognizable anymore. In models treated with neat DEEA, only cell debris remained, and single cells could not be distinguished anymore. For BAC, histology revealed a stratified epithelium after the application of 0.01%, but a thinner epithelium with less cornification and pyknotic nuclei in the other conditions.

## Discussion

Tissue engineering is a promising approach to develop test systems for toxicological evaluations. Here, the eye irritation potential of substances in dilution was assessed using a cornea-like epithelium. We found that the concentration which can be applied to a model without impairing it differs greatly between chemicals. This underpins the necessity for practical testing, as the theoretical classification can fail to predict the hazard potential.

The 3D tissue models used as a test system in this study are based on human epithelial keratinocytes. Being primary cells from different donors, they are advantageous over cell lines in predicting the in vivo situation^14,15^. Further, the use of human cells enables the recognition of species-specific reactions to chemicals. Although originating from the skin, the cells were previously shown to be a suitable model to predict corneal irritation. They were compared to models based on corneal cells and further accepted in official test guidelines^5,16^. As the EIT is conducted 11 days after the set-up of the 3D model, the epithelium is not yet keratinized, displaying a vulnerability comparable to the cornea. The cornification in the presented histological images was built up from differentiating cells after EIT.

For chemicals causing serious eye damage, Annex 1 of the CLP Regulation sets the following concentration limits: If the dilution contains ≥ 1% but < 3% of the chemical, it is considered a category 2 dilution. If the chemical is present in concentrations ≥ 3%, the dilution falls in category 1. Nevertheless, there are exceptions and existing data regarding the specific chemical needs to be taken into account. In the C&L Inventory, companies can provide information on specific concentration limits (SCLs) for chemicals. Although not reviewed, this database can give further orientation for the labeling of dilutions. If the SCLs for a chemical are known, it overrules the general concentration limits given in the CLP Regulation.

Of the four chemicals tested in this study, THF was the least irritative. Within the tested conditions, only the neat substance affected the viability of RCE models, while the models treated with dilutions proved viable in Histology and MTT. The sensitive TEER measurement showed a slightly lower barrier function after treatment with 5% THF, compared to models treated with 1%. Still, both dilutions would not fall in any GHS category according to our categorization. Here, the theoretical categorization might overpredict the toxicity. Although labeled as a category 1 substance by the ECHA, some studies found THF to be only moderately irritative when applied to rabbit eyes^9^. In line with that, THF showed milder toxicity in our experiments, if compared to other category 1 substances. The Classification and Labeling (C&L) Inventory of the ECHA states an SCL of concentration (C) ≥ 25% for eye irritation category 2 by tetrahydrofuran^17^.Following the CLP Regulation, 1% THF would be considered a category 2, 5% THF a category 1. Our results support the data given in the C&L Inventory. The general limits of the CLP Regulation would, in this case, overpredict the toxicity of THF dilutions.

For AA, we observed no irritative effect for dilutions by histology and MTT, yet impedance spectroscopy showed a transient impairment of the barrier function after the application of 5% AA. This prompts a classification of 5% AA as a category 2 substance. 1% AA does not fall in any GHS category. Other studies demonstrated that 3% AA causes slight irritation, while 10% AA causes irreversible eye damage^18–20^. Combining our results with this data, a minimal concentration of 1—3% AA is needed for reversible eye irritation and a minimum concentration of 5–10% AA for irreversible eye damage. The C&L Inventory lists an SCL of 10% ≤ C < 25%. In concentrations ≥ 25%, AA is stated to be skin corrosive and thus exerts severe damage to both eyes and skin. Effects of AA in concentrations < 10% are not reported^21^. As for THF, both the C&L Inventory and our data suggest a lower toxicity of AA than predicted by the CLP Regulation.

The irritation caused by 5% DEEA was permanent as detected by impedance spectroscopy. In MTT some models were still viable, the high standard variation indicates differences between the models of different donors. By histology, destruction of the tissue could be visualized. Only TEER measurement could provide reliable recognition of the caused irritation. According to our prediction model, GHS labeling is thus recommended for products containing ≥ 5% DEEA. No effects were detected after EIT with 1% DEEA. Thus, it is not subject to labeling. Unfortunately, no similar studies on the toxicity of DEEA in dilutions are published and thus our data could not be compared with results from other methods. No SCL is mentioned in the C&L Inventory database^22^. Here, the theoretical categorization following the CLP Regulation would lead to the same result as our test method.

BAC damaged the epithelium even in dilutions of 0.1% and would hence fall in category 1. Our results are in line with literature showing the toxicity of BAC in concentrations < 1%^23,24^. Research on the mode of action in conjunctival cells showed the induction of apoptosis by BAC^25^. Cell death was confirmed in our experiments by histology and viability assay. In eyedrops, BAC is present in lower concentrations than tested in this EIT, mostly < 0.1%^26^. No statement was made in the C&L Inventory about SCLs of BAC. The general concentration limits of the CLP Regulation would clearly underpredict the toxicity, as it does not respect chemicals in concentrations < 1%. Nevertheless, the Regulation also states that upon knowledge of a toxic effect in concentrations < 1%, this must be taken into account and the respective solution must be labeled accordingly.

The comparison of the diluted substances underpins the necessity of in vitro testing. No general statement can be made to predict the critical concentration for the toxicity of a diluted substance. The main focus of GHS classification is still based on neat chemicals using the Draize database^7^. There is only few comprehensive animal reference data to validate the in vitro model for dilutions. Still, for neat substances, the results based on the TEER-based prediction model were in line with the in vivo data and a good transferability can therefore be assumed. Furthermore, our results are corroborated by the available SCLs, or, where no SCL is given, with the theoretical classification of the CLP Regulation. Yet for many chemicals, no SCLs are known. Relying on the general concentration limits might lead to a wrong classification.

In comparison to the methods used in other in vitro eye irritation tests, TEER measurements are a non-invasive analysis. The aspect of long-term regeneration, as a crucial feature of category 2 substances, can thus be taken into account. To face the lack of comprehensive reference data, especially for dilutions, long-term characterization of tissue integrity would extend the range of characterization opportunities to improve the prediction of eye irritations. Due to the high sensitivity of impedance spectroscopy, it can also detect differences between models based on different donors. This can lead to higher standard deviations if, as in this study, biological replicates are used.

To summarize, our research depicts the effects of chemicals in dilutions on a cornea-like model. The eye irritation potential of diluted chemicals revealed limitations of the current theoretical guidelines. Validation of the presented test method by the Organization for Economic Co-operation and Development (OECD) is therefore intended. This would set another example of a non-animal method included in regulatory law. To further simplify the labeling of both industrial and consumer products, testing of solutions containing various chemicals is aimed for in the future. The opportunity to test complex solutions in vitro would contribute to consumer safety.

## Materials and methods

### Human material

Human material was obtained by the University Hospital Würzburg, Germany. All samples were acquired after informed consent of the patients and their legal guardians according to the Declaration of Helsinki. The use of biopsies was approved by the ethics commission of University Würzburg 182/10 and 280/18sc.

### Cell isolation and culture

To generate the reconstructed cornea-like epithelium (RCE) models human epithelial keratinocytes (HEKs) were isolated from foreskin biopsies of 2–5-year-old donors as described previously^5^. In brief, the tissue was washed with Phosphate-buffered Saline supplemented with Calcium and Magnesium (PBS^+^, Sigma-Aldrich, Germany). Connective and fat tissue was separated from the skin using a scalpel and subsequently discarded. The foreskin was dissected in stripes and dispase (Life Technologies, USA) was employed to detach the epidermis from the dermis. Therefore, the foreskin tissue was incubated in 2 U/ml of dispase for 16–18 h at 4 °C, followed by separation of the two layers with forceps. The epidermal part was transferred to a fresh petri dish with PBS^+^ and further cut into smaller pieces. The epidermis was incubated in a 10 ml solution of 0.05% Trypsin–EDTA (Life Technologies, USA) in Phosphate-buffered Saline w/o calcium and magnesium (PBS^-^, Sigma Aldrich, Germany) for 5 min to achieve a single-cell suspension. The cell suspension was filtered using a 100 µm cell strainer (Greiner Bio-One, Austria) and centrifuged at 300 g for 5 min. The cells were resuspended in E1 Medium (see Table 1) and seeded at a density of 4000 cells/cm^2^. The medium was replaced every 2–3 days. When 80–90% confluency was reached, cells were passaged or cryo-conserved.

**Table 1:** Cell culture media.

Medium	Composition
E1	EpiLife™ medium (Life Technologies, USA) + 1% human keratinocyte growth supplement (Life Technologies, USA) + 1% Penicillin/Streptomycin (Sigma-Aldrich, Germany)
E2	E1 medium + 1.44 mMCaCl_2_ (Sigma-Aldrich, Germany)
E3	E2 medium + 73 µg/ml Ascorbyl-2-phosphate (Sigma-Aldrich, Germany) + 10 ng/ml keratinocyte growth factor (KGF) (Life Technologies, USA)

Cells were tested for mycoplasma several times a year using a mycoplasma detection kit (Minerva Biolabs, Germany).

### Generation of RCE models

RCE models were generated following a previously published protocol^5^. Briefly, HEKs were used at passage 3 and cultured to a confluency of around 80%. The cells were detached by incubation with Accutase^®^ for 10 min. The cell suspension was centrifuged for 5 min at 300 g and the pellet was resuspended in E2 medium. The RCE models were cultured in inserts with a polycarbonate membrane with 0.4 µm pores and a culture area of approximately 0.6 cm^2^ (BRAND, Germany). 3 × 10^5^ cells in a volume of 300 µl E2 medium were seeded per insert and incubated under standard culture conditions (37 °C, 95% humidity, and 5% CO_2_) for 2 h. 1.4 ml of E2 medium were added to each well outside of the insert. The medium within the insert was removed 24 h after submers culture and the medium in the well was replaced by 1.4 ml of E3 medium to create an airlift culture. During subsequent cultivation, the medium was replaced every 2–3 days.

### Eye irritation test (EIT)

**Figure 5:**
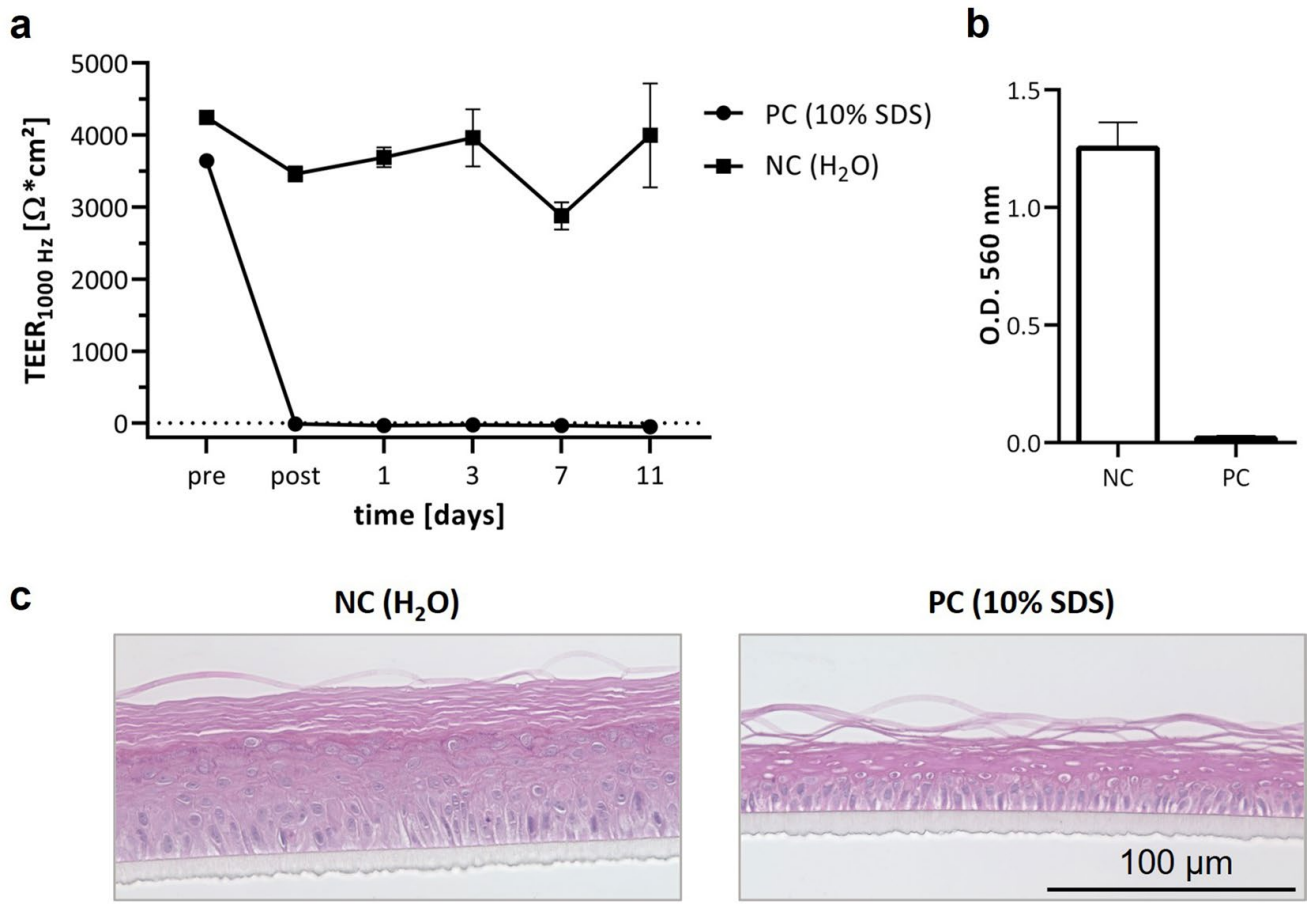
Eye irritation can be depicted by impedance spectroscopy, viability assay, and histology. (**a**) Impedance spectroscopy, depicted as TEER-values at 1000 Hz. TEER-values of two models of one donor were measured for each control, and means with standard deviation of three different donors are shown. Measurements were performed before and after EIT, and on day 1, 3, 7, and 11 after EIT. (**b**) MTT assay on day 11 after EIT, one model was used in technical duplicates. Means are shown with standard deviation of three donors (**c**) H&E staining images of RCE models on day 7 after EIT. One model was used per condition of each EIT, Histology.

For the eye irritation test, RCE models were employed on day 11 of culture before keratinization. The test protocol was performed based on the OECD test guideline 492^27^. In short, the RCE models were moisturized with 20 µl PBS^—^for 30 min before application of 50 µl of the test substance for 30 min. The substance was removed by three washing steps with PBS^-^ and the models were placed in a 12-well plate with 5 ml EpiLife™ supplemented with 1.44 mM CaCl_2_ per well for 12 min. Normal culturing conditions were applied for further 120 min. In addition to the protocol described in the OECD test guideline 492, we included TEER measurements as non-invasive test method. Further, we performed MTT on day 11 instead of directly after EIT. The prediction model based on the impedance data uses the mean values of the biological replicates and is described in detail in previous publication^5^ (Fig. 1). Each EIT was performed on models of three different donors in individual experiments. Per donor and diluted chemical, four models were used. TEER measurements were performed on two models, one model was used in technical duplicates for MTT measurements, and one model was used for histology. The same conditions were used for the negative and positive controls. As positive control, we used 10% Sodiumdodecylsulfate (SDS, Sigma Aldrich, Germany), as negative control deionized water. The controls are shown in Fig. 5. The four test substances tetrahydrofuran, acetic acid, diethylethanolamine, and benzalkonium chloride are liquid substances of GHS category 1 and were obtained from Sigma-Aldrich, Germany. The substances were diluted in deionized water to the respective concentrations or used neatly at 100%.

To picture the morphology of the RCE models, histology was performed as described before^5^ on day 7 after EIT. Briefly, the models were fixed in ROTI®Histofix (Carl Roth, Germany), embedded in paraffin, cross-sectioned in a width of 3.5 µm, and stained with hematoxylin and eosin (H&E; Morphisto, Germany). Images were acquired with a Keyence microscope Biorevo BZ-9000 (Keyence, Japan).

### MTT assay

The viability of cells can be determined with the formazan dye 3-(4,5-Dimethylthiazol-2-yl)-2,5-diphenyltetrazoliumbromid (MTT). The protocol was performed as described in Ref.^28^ for models on day 11 after EIT. In short, 200 µl of 1 mg/ml MTT (Sigma Aldrich, Germany) in PBS^+^ were used per well and incubated with the model for 3 h at 37 °C. The dye was solubilized in 2 ml isopropanol by gentle shaking for 30 min. 200 µl of the solution were used for analysis. The extinction of two technical duplicates was measured for each model at 570 nm using a Tecan plate reader M Infinite nano (Tecan, Switzerland).

### Impedance spectroscopy

The barrier function of RCE models was quantified by impedance spectroscopy. Impedance was measured before and after EIT (day 0), as well as on day 1, 3, 7, and 11 after EIT. The procedure is described in Ref.^4^. Briefly, models were transferred to a 24 well measuring plate (BRAND GmbH + Co KG, Germany) and 600 µl EpiLife™ medium containing 0.48% 300 mM CaCl_2_-Solution and 1% P/S were added in the apical and the basolateral compartment. Titanium nitride (TiN)-electrodes, integrated in a 24-well plate format, were used to measure the impedance and are described in Ref.^29^.The used device was the impedance analyzer LCR HiTESTER 3522-50 (HIOKI, Japan). The transepithelial electrical resistance (TEER) was measured at 1000 Hz (TEER_1000 Hz_) as described in detail in Ref.^4^. The TEER values of treated tissue models were normalized to the mean value of the negative control on each respective day. To ensure barrier quality, TEER values ranged from 500 to 5000 Ohm × cm^2^ prior to the test were used for the application.

### Data analysis

Data was analyzed using GraphPad Prism 8 (Graphpad Software, USA). Statistical analysis included a two-way anova for impedance, followed by Tukey’s multiple comparisons test.

## Data Availability

The datasets generated during and/or analyzed during the current study are available from the corresponding author on reasonable request.
